# Signaling Pathways Potentially Responsible for Foam Cell Formation: Cholesterol Accumulation or Inflammatory Response—What is First?

**DOI:** 10.3390/ijms21082716

**Published:** 2020-04-14

**Authors:** Alexander N. Orekhov, Vasily N. Sukhorukov, Nikita G. Nikiforov, Marina V. Kubekina, Igor A. Sobenin, Kathy K. Foxx, Sergey Pintus, Philip Stegmaier, Daria Stelmashenko, Alexander Kel, Anastasia V. Poznyak, Wei-Kai Wu, Artem S. Kasianov, Vsevolod Y. Makeev, Ichiro Manabe, Yumiko Oishi

**Affiliations:** 1 Institute of General Pathology and Pathophysiology, 8 Baltiiskaya Street, 125315 Moscow, Russia; vnsukhorukov@gmail.com (V.N.S.); nikiforov.mipt@googlemail.com (N.G.N.); igor.sobenin@gmail.com (I.A.S.); 2 Institute of Human Morphology, 3 Tsyurupa Street, 117418 Moscow, Russia; 3 Institute of Experimental Cardiology, National Medical Research Center of Cardiology, 15A 3-rd Cherepkovskaya Street, 121552 Moscow, Russia; 4 Centre of Collective Usage, Institute of Gene Biology, Russian Academy of Sciences, 34/5 Vavilova Street, 119334 Moscow, Russia; marykumy@gmail.co; 5 Kalen Biomedical, LLC, Montgomery Village, MD 20886, USA; info@kalenbiomed.com; 6 BIOSOFT.RU, LLC, 630090 Novosibirsk, Russia; sspintus@developmentontheedge.com (S.P.); dashanikolaeva@gmail.com (D.S.); alexander.kel2@gmail.com (A.K.); 7 Institute of Computational Technologies, 630090 Novosibirsk, Russia; 8 geneXplain GmbH, 38302 Wolfenbüttel, Germany; info@geneXplain.com; 9 Institute of Chemical Biology and Fundamental Medicine, 630090 Novosibirsk, Russia; 10 Institute for Atherosclerosis Research, Skolkovo Innovative Center, 121609 Moscow, Russia; 11 Department of Internal Medicine, National Taiwan University Hospital, Bei-Hu Branch, Taipei 10002, Taiwan; weikaiwu0115@gmail.com; 12 Institute for Information Transmission Problems Russian Academy of Sciences, Bolshoy Karetny per. 19, build.1, 127051 Moscow, Russia; a.kasianov@skoltech.ru; 13 Vavilov Institute of General Genetics Russian Academy of Sciences, Gubkina str. 3, 119333 Moscow, Russia; vsevolod.makeev@gmail.com; 14 Moscow Institute of physics and technology, 9 Institutskiy per., 141701 Dolgoprudny, Moscow Region, Russia; 15 Engelhardt Institute of Molecular Biology of Russian Academy of Sciences, 32 Vavilova Street, 119991 Moscow, Russia; 16 Department of Aging Research, Graduate school of Medicine, Chiba University, Chiba 263-8522, Japan; manabe-tky@umin.ac.jp; 17 Department of Biochemistry & Molecular Biology, Nippon Medical School, Tokyo 113-8602, Japan; yuooishi@gmail.com

**Keywords:** atherosclerosis, foam cells, gene knockdown, inflammatory molecules, latex beads, macrophages, modified low-density lipoprotein, phagocytosis, self-association, signaling pathways, transcriptome analysis

## Abstract

Accumulation of lipid-laden (foam) cells in the arterial wall is known to be the earliest step in the pathogenesis of atherosclerosis. There is almost no doubt that atherogenic modified low-density lipoproteins (LDL) are the main sources of accumulating lipids in foam cells. Atherogenic modified LDL are taken up by arterial cells, such as macrophages, pericytes, and smooth muscle cells in an unregulated manner bypassing the LDL receptor. The present study was conducted to reveal possible common mechanisms in the interaction of macrophages with associates of modified LDL and non-lipid latex particles of a similar size. To determine regulatory pathways that are potentially responsible for cholesterol accumulation in human macrophages after the exposure to naturally occurring atherogenic or artificially modified LDL, we used transcriptome analysis. Previous studies of our group demonstrated that any type of LDL modification facilitates the self-association of lipoprotein particles. The size of such self-associates hinders their interaction with a specific LDL receptor. As a result, self-associates are taken up by nonspecific phagocytosis bypassing the LDL receptor. That is why we used latex beads as a stimulator of macrophage phagocytotic activity. We revealed at least 12 signaling pathways that were regulated by the interaction of macrophages with the multiple-modified atherogenic naturally occurring LDL and with latex beads in a similar manner. Therefore, modified LDL was shown to stimulate phagocytosis through the upregulation of certain genes. We have identified at least three genes (*F2RL1*, *EIF2AK3*, and *IL15*) encoding inflammatory molecules and associated with signaling pathways that were upregulated in response to the interaction of modified LDL with macrophages. Knockdown of two of these genes, *EIF2AK3* and *IL15*, completely suppressed cholesterol accumulation in macrophages. Correspondingly, the upregulation of *EIF2AK3* and *IL15* promoted cholesterol accumulation. These data confirmed our hypothesis of the following chain of events in atherosclerosis: LDL particles undergo atherogenic modification; this is accompanied by the formation of self-associates; large LDL associates stimulate phagocytosis; as a result of phagocytosis stimulation, pro-inflammatory molecules are secreted; these molecules cause or at least contribute to the accumulation of intracellular cholesterol. This chain of events may explain the relationship between cholesterol accumulation and inflammation. The primary sequence of events in this chain is related to inflammatory response rather than cholesterol accumulation.

## 1. Introduction

The accumulation of lipid-laden (foam) cells in the arterial wall is the earliest step in the pathogenesis of atherosclerosis [1,2,3]. It is generally accepted that the main sources of accumulating lipids (mostly cholesterol and cholesteryl esters) in foam cells are atherogenic modified low-density lipoproteins (LDL). Arterial cells (macrophages, pericytes, and smooth muscle cells) take up these particles in an unregulated manner bypassing the specialized LDL receptor [3,4,5]. The mechanism of interaction of native (unmodified) LDL with a specific cell receptor is currently well known [5,6,7,8]. This interaction does not lead to excessive deposition of intracellular lipids since the lipid components of LDL are utilized by cells, and the excess is removed. Thus, native LDL is not atherogenic because it does not induce the formation of foam cells.

On the other hand, it was found that chemically modified LDL had atherogenic properties, hence, caused lipid accumulation in cultured cells. Atherogenic modifications of LDL that have been described so far include acetylation, desialylation, maleylation, succinylation, oxidation by ions of metals of variable valence, treatment with formaldehyde, malondialdehyde, phospholipase A2, C, and D, etc. [9,10,11,12,13,14,15,16,17].

Multiple modified LDL species can be found in the circulation of atherosclerotic patients [3,16,17]. Noteworthy, atherogenic modification of LDL is not limited to oxidation. Misconceptions about the key role of oxidized LDL in the formation of foam cells are widespread. However, alteration of other physical–chemical characteristics of the lipid, protein, and glycoconjugate moieties of lipoprotein particles have been revealed [16,17]. Oxidation appears to be neither the primary nor the only atherogenic modification of LDL. The key mechanism of interaction between modified LDL and macrophages are recognition and internalization via scavenger receptors. This leads to lipid accumulation and foam cell formation [3,16,17]. Notably, macrophages are not the only source of foam cells. Resident and migrating smooth muscle cells and pericyte-like cells can also uptake LDL particles and transform into foam cells [3]. Pro-inflammatory cytokines are released, such as IL-8, IL-1, and IFN-γ, that contribute to the recruitment of more and more monocytes and macrophages, thus trigger plaque growth [18]. 

After being formed, foam cells accumulate in the arterial intima and form a fatty streak that is an initial stage of atherosclerotic plaque development. [19]. Further plaque formation contributes to the release of proteases that degrade the extracellular matrix. Collagen and elastin, derived from this cleavage, are responsible for the fibrous cap formation that encloses the necrotic core. Cells within the necrotic core die and release their cellular content, which results in the formation of the lipid-rich necrotic core. At this stage, the plaque is called intermediate. Further plaque development contributes to the enhancement of these processes and the growth of the necrotic environment [18]. 

In this study, we used transcriptome analysis (RNA-seq) for identifying genes and regulatory pathways potentially responsible for cholesterol accumulation in human monocyte-derived macrophages exposed to artificially modified or naturally occurring circulating atherogenic LDL particles. The LDL samples tested in the study included native LDL, multiple modified LDL isolated from the blood of atherosclerotic patients, and LDL modified in vitro by acetylation, oxidation, and desialylation. In addition, we used latex beads to stimulate macrophage phagocytic activity. In our previous studies, we demonstrated that any type of LDL modification facilitated the self-association of lipoprotein particles [19]. The increased size of such self-associates precluded their proper interaction with a specific LDL receptor. As a result, they were taken up by nonspecific phagocytosis bypassing the LDL receptor. In the present study, we aimed to establish the possible common mechanisms in the interaction of macrophages with modified LDL self-associates and non-lipid latex particles of a similar size.

## 2. Results

### 2.1. Sample Preparation

**Figure 1:**
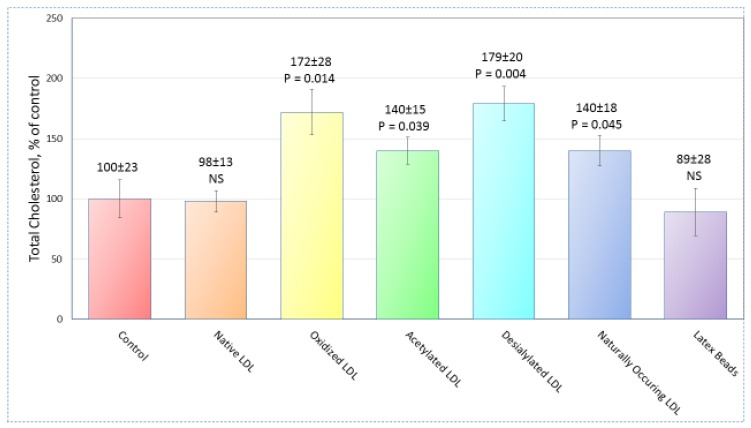
Cholesterol content in cultured macrophages. Total cholesterol (nmol/mg protein) in cultured macrophages was measured after treatment with different low-density lipoproteins (LDL) samples and latex beads. Data are represented as mean ± standard deviation and *p*-value vs. control. NS, not significant differences from control. Differences between control, LDL preparations, and latex beads were identified using the paired sample *t*-test with 21.0 IBM SPSS Statistics.

In accordance with our expectations, the total cholesterol content in cultured macrophages was not influenced by the low concentrations of both native LDL (50 µg protein/mL) and latex beads. On the other hand, the addition of each of acetylated, oxidized, and desialylated LDL resulted in significant enhancement of the total cellular cholesterol. Data on the total cholesterol content measurement after 24 h are summarized in Figure 1. All tests were performed triply. High-throughput sequencing of mRNA obtained from the cells treated with naturally occurring, modified LDL, or latex beads was performed on 21 samples from 7 groups, as shown in Figure 1 (monocytes/macrophages were obtained from 3 different donors in each group).

### 2.2. Search for Signaling Pathways

RNA sequence data were analyzed using “upstream analysis” [20,21]. Our aim was to identify regulatory networks that may affect the activity of transcription factors (TFs) that, in turn, control differential expression of the genes. This signaling pathway possibly controls foam cell formation. The upstream analysis used in this study consisted of three consequent steps: identification of DEGs, analysis of promoters and TFs of DEGs, and search for potential master regulators. In this work, we did not identify master regulators but limited ourselves to identifying signaling pathways.

**Figure 2:**
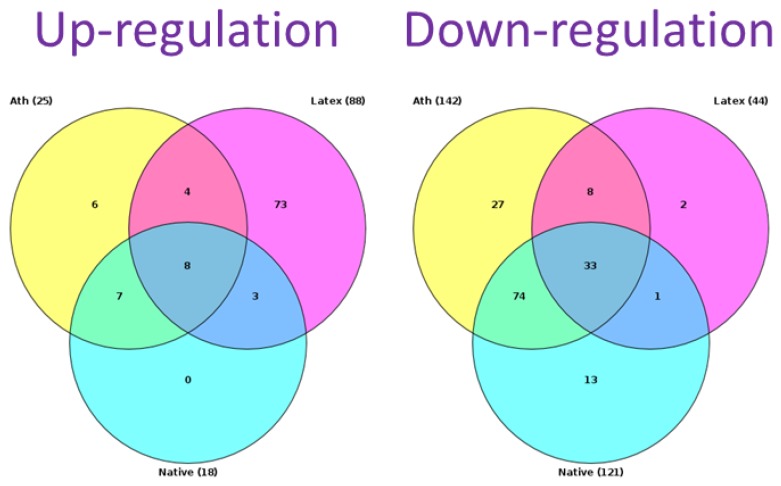
Venn diagram of signaling pathways. “Native” stands for pathways that were identified by incubation of cultured monocyte-derived macrophages with naturally occurring LDL; “Ath” stands for pathways that were identified by incubation of cultured monocyte-derived macrophages with naturally occurring LDL particles obtained from the blood of individuals with atherosclerosis; “Latex” stands for pathways that were identified by incubation of monocyte-derived macrophages with latex beads. The number of revealed pathways is shown in brackets.

We identified signaling pathways that were upregulated and downregulated in macrophages in response to different lipoprotein samples. In this study, we did not consider native LDL that did not cause lipid accumulation. Thus, we considered only those pathways that were regulated differently from control samples (native LDL). Figure 2 presents the Venn diagram, which compares the pathways of naturally occurring LDL and latex beads. Of 88 upregulated and 44 downregulated pathways identified for latex beads, 77 and 10 differed from the control, respectively. There were signaling pathways that changed similarly in response to latex beads and modified LDL. Comparison of pathways whose regulation was changed unidirectionally revealed the pathways that were similarly regulated for naturally occurring LDL and latex beads. Among these signaling pathways, four underwent upregulation and eight, downregulation. 

Table 1 presents the signaling pathways regulated by the interaction of macrophages with latex beads and modified LDL. The pathways that were regulated unidirectionally (up or down) in the case of naturally occurring LDL and lipoproteins artificially modified in vitro (desialylated, acetylated, and oxidized) are highlighted in different colors. Signaling pathways, adjustable unidirectionally in all four types of modifications, are highlighted in green. This pathway turned out to be one, namely the “TLR9 pathway”. Signaling pathways, the direction of regulation of which coincides with the three types of modifications, are highlighted in cyan. Finally, the signal pathways coinciding only in the case of two modifications, are highlighted in yellow. Pathways regulated by LDL modified in vitro were compared with pathways regulated by circulating naturally occurring LDL. All signaling pathways regulated by naturally occurring LDL have the same regulation with at least one type of in vitro modification. A complete coincidence was found for the signaling pathways downregulated by naturally occurring and desialylated LDL. For upregulation, there were only two matches, “neurotrophic signaling” and “TLR9 pathway”. Thus, the total number of matches for naturally occurring and desialylated LDL was 10 out of 12.

Two other types of atherogenic modification (acetylation and oxidation) were dramatically different from naturally occurring LDL. In the case of acetylated LDL, all four upregulated signaling pathways characteristic of naturally occurring LDL coincided. However, another 29 upregulated signaling pathways that were not regulated by circulating atherogenic LDL were also detected for acetylated LDL. Acetylated LDL downregulated only one signaling pathway, which coincided with that of naturally occurring LDL. The total number of matches for naturally occurring and acetylated LDL was 5 out of 12. Even fewer matches were found for oxidized LDL that did not cause downregulation at all. Only two upregulated pathways coincided (“TLR2-mediated signaling” and “TLR9 pathway”). Thus, only 2 out of 12 matches were found for naturally occurring and oxidized LDL.

### 2.3. The Relationship between Key Genes and Signaling Pathways

We found that at least 12 signaling pathways were similarly regulated by naturally occurring LDL and latex beads. Stimulation of phagocytosis can be regarded as a likely link between the two stimuli. Modified LDL forms self-associates that are comparable in size to latex beads and cannot interact with a specific LDL receptor but are taken up by the cell through phagocytosis [22,23,24]. Stimulation of phagocytosis is a trigger for the macrophage pro-inflammatory response accompanied by the secretion of pro-inflammatory cytokines.

**Figure 3:**
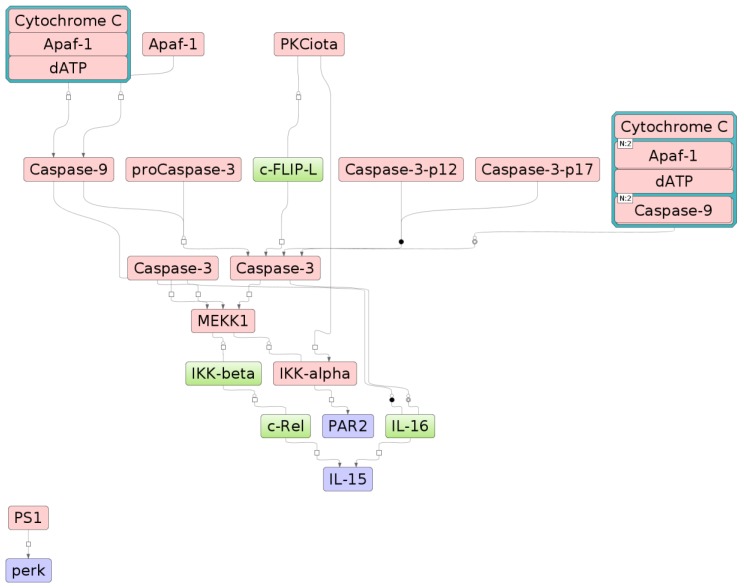
The signal transduction pathway is represented as a network diagram shown in SBGN format (Systems biology graphical notations). Proteins that are involved in the pathway are shown as green bars with names of the proteins. Complexes of the proteins are shown by combining several proteins in one node. Nodes on the diagram are connected by lines that represent reactions (binding, phosphorylation, and other types of signaling reactions). Relationship between the components of the neurotrophic signaling pathway with the *F2RL1* (PAR2), *EIF2AK3* (PERK), and *IL15* genes. The proteins encoded by the genes of the master regulators PAR2, PERK, and IL15 are highlighted in blue. The components of the neurotrophic signaling pathway are highlighted in red. The blue border around the input points from neurotrophic signaling.

In our previous studies, we identified key genes (master regulators) associated with the accumulation of intracellular cholesterol caused by modified lipoproteins [25]. We found that some of these genes, in particular, the *F2RL1* (encoding for PAR2), *EIF2AK3* (PERK), and *IL15*, were related to neurotrophic signaling, TLR2, and TLR9 signaling pathways identified in this study. To establish the relationship between master regulators and signaling pathways, we performed an “upward” test of the signal transmission network based on the information from the TRANSPATH^®^ database using the algorithm described previously [26]. Starting from the targeted proteins encoded by the *F2RL1* (PAR2), *EIF2AK3* (PERK), and *IL15* genes, the algorithm moves up the signaling network and finds such key components of it from which targeted proteins can be reached in a minimum number of steps. Next, we combined the list of the identified key network components with lists of proteins that made up the different canonical signaling pathways in the cells. For example, for the neurotrophic signaling pathway, a statistically significant number of such intersecting components encoded by the nine genes *APAF1, CASP3, CASP9, CHUK, CYCS, IKBKB, MAP3K1, PRKCI, PSEN1* (Figure 3 and Figure S2) was revealed. Figure S2 shows the relationship between the proteins encoded by the *F2RL1* (PAR2), *EIF2AK3* (PERK), and *IL15* genes (highlighted in blue) with key components of the signal network (highlighted in red). It is worth clarifying here that the *IL15*, *EIF2AK3* (PERK), *F2RL1* (PAR2) genes by themselves are not part of the neurotrophic signaling pathway but are linked with molecules that are part of this pathway. This means that activation of the identified signal transduction pathways may lead to the consequent activation of the master regulators of our interest associated with the accumulation of intracellular cholesterol caused by modified lipoproteins. Molecules that are part of the neurotrophic signaling are highlighted in red in the diagram; *IL15*, *EIF2AK3* (PERK), *F2RL1* (PAR2) are shown in blue. These genes are known to be associated with the inflammatory response [27,28,29,30,31,32].

### 2.4. Knockdown of Key Genes

We next tested whether the *F2RL1*, *EIF2AK3*, and *IL15* genes could be involved in the accumulation of intracellular cholesterol. To this end, we knocked down these genes in primary macrophages derived from human monocytes. Cells were cultured for 24 h with the addition of atherogenic naturally occurring LDL. We showed the significant impact of atherogenic LDL on the increase in cholesterol accumulation in cultured cells. In contrast, the knockdown of the *EIF2AK3* and *IL15* genes appeared to inhibit it (see Table 2) effectively. However, the same effect was not shown for the knockdown of *F2RL1*.

It should be noted that the studied genes are u-regulated during the accumulation of intracellular cholesterol caused by modified LDL [25]. It can, therefore, be assumed that the *EIF2AK3* and *IL15* genes are directly involved in the accumulation of cholesterol, contributing to an increase in its content during the interaction of cells with modified LDL, since the knockdown of these genes completely suppressed the accumulation.

## 3. Discussion

According to current understanding, the initial stages of atherosclerosis development are dependent on two processes: accumulation of intracellular lipids (mainly cholesterol and its esters) and the inflammatory response of the resident arterial cells accompanied by the recruitment of circulating inflammatory cells into the subendothelial space of arterial intima where they differentiate into macrophages of the vascular wall [33]. We have previously shown that lipid accumulation within the arterial cells is the trigger that initiates the activation of cellular functions associated with atherogenesis [34]. It is generally accepted that the source of cholesterol accumulation is modified LDL [35,36]. Atherogenic multiple-modified LDL that can cause the accumulation of intracellular lipids [3,16,17] circulate in the blood of atherosclerotic patients.

It was widely believed that it is the accumulation of intracellular lipids that stimulates the pro-inflammatory response [37,38,39,40,41,42,43,44]. However, it remains unknown whether intracellular lipid metabolism and the immune response are independently regulated by modified LDL or regulation of one process drives changes in the other. These two processes do interact [45], but it remains uncertain regarding the true primary driver in this interaction. Indeed, changes in cholesterol metabolism can affect inflammatory responses of macrophages [37,38,39,40,43,46,47,48]. However, we recently revealed ten genes that may be the key regulators of foam cell formation [25]. Surprisingly, seven of these ten genes belong to the inflammatory pathway, and none of them belonged to the cholesterol metabolism pathways. This suggests that the accumulation of cholesterol is not the primary event triggering the immune response, but rather the pro-inflammatory immune response triggers the accumulation of intracellular cholesterol, or at least contributes to this process.

**Figure 4:**
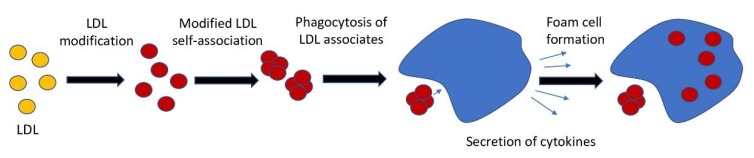
The sequence of events leading to the accumulation of intracellular cholesterol (from [49]).

In this study, we hypothesized that phagocytosis is the key event that combines the accumulation of cholesterol and the inflammatory response. According to the classical concept, phagocytosis of the pathogen is precisely the process from which the innate immunity reaction begins. The next event is the secretion of pro-inflammatory molecules recruiting circulating inflammatory cells into the focus of possible inflammation. Resident subendothelial cells of the arterial wall can respond to particles of modified LDL by stimulating phagocytosis since these particles tend to self-associate [19,24], and the resulting associates are quite comparable in size to the pathogen (e.g., bacteria) (Figure 4). We propose the following sequence of events in atherosclerosis initiation: LDL particles undergo atherogenic modification; this is accompanied by the formation of self-associates; large LDL self-associates stimulate phagocytosis; pro-inflammatory molecules are secreted as a result of phagocytosis stimulation; these molecules cause or contribute to the accumulation of intracellular cholesterol. This hypothesis allows us to explain how the inflammatory response and the accumulation of cholesterol are related. At the same time, it becomes obvious that the primary in this sequence of events is not the accumulation of cholesterol but an inflammatory response.

We analyzed 12 signaling pathways that were found to be similarly regulated by the interaction of macrophages with the multiple-modified atherogenic naturally occurring LDL and with latex beads, which are the classic stimulant of phagocytosis. Thus, we confirmed our aforementioned hypothesis. The forms of in vitro artificially modified LDL were also studied. These forms also had common signaling pathways with latex beads and naturally occurring LDL. It turned out that in terms of signaling pathway regulation, desialylated LDL is most similar to latex beads and naturally occurring LDL. The oxidized and acetylated LDL, widely used in research, had much fewer similarities. We can conclude that these forms of modified LDL are a poor model, at least for the study of gene regulation.

Thus, we showed that modified LDL triggered the genetic regulation characteristics of the stimulation of phagocytosis. To confirm our hypothesis, it was essential to show that as a result of this regulation, inflammatory molecules are formed that can affect the accumulation of intracellular cholesterol. We have identified at least three genes (*F2RL1*, *EIF2AK3*, and *IL15*) encoding inflammatory molecules and associated with the identified signaling pathways. Interaction of macrophages with modified LDL, led to the upregulation of these genes [25]. For two of these genes, EIF2AK3 and IL15, knockdown completely suppressed the accumulation of cholesterol in macrophages. It follows that upregulation of at least genes *EIF2AK3* and *IL15* promotes the accumulation of cholesterol. Indeed, EIF2AK3 directly involved in cholesterol accumulation as it upregulated CD36 and SRA and downregulated on ABCA1, ABCG1, and SRB1 expressions [50]. The role of IL-15 in lipid accumulation is not clear and requires further investigation. Currently, it is known that IL-15 inhibition promotes attenuation of atherosclerotic lesions [51], and this interleukin is involved in inflammation in adipose tissues leading to obesity-associated metabolic syndrome [52]. Activation of the PAR2 receptor led to the upregulation of inflammatory gene expression and enhanced lipid accumulation in macrophages [53]. Apparently, inhibition of expression of F2RL1 did not prevent lipid accumulation, as we have shown. Moreover, IL-15 and PAR2 (F2RL1) may be involved in phagocytosis of aggregated LDL. Specifically, IL-15 increased phagocytosis alone [54], and together with GM-CSF [55], the inhibition of PAR2 by antibodies [56] or knockout leads to inefficient phagocytosis [57]. However, our data confirmed that only IL-15 might influence cholesterol accumulation through the phagocytosis mechanism. Taken together, these data confirmed our hypothesis, schematically presented in Figure 4. For the final proof of the validity of this hypothesis, extensive studies of the effect of inflammatory molecules on the accumulation of lipids in cells are needed.

## 4. Materials and Methods 

The study was conducted in accordance with the Helsinki Declaration and was approved by the institutional Ethics Committee (Institute for Atherosclerosis Research, Moscow, Russia). 

### 4.1. Lipoproteins and Latex Beads

Latex beads sized 1.1 μm (LB11, Merck, Darmstadt, Germany) were used to activate unspecific phagocytosis. Native human LDL with electrophoretic mobility 105 ± 1 pixels and modified LDL (oxidized with electrophoretic mobility 491 ± 5 pixels and acetylated with electrophoretic mobility 477 ± 5 pixels) were obtained from Kalen Biomedical (Montgomery Village, MD, USA). Preparing of desialylated LDL was performed according to the previous description [58]. Normalized by total protein content, 2 mg/mL native LDL were treated with neuraminidase immobilized on agarose carrier (40 μU/mL) to reduce the sialic acid content by relatively 70%. This treatment lasted 2 h at 37 °C. After that, agarose beads were removed from samples by the centrifugation for 10 min at 2500 rpm. Then samples were dialyzed against phosphate-buffered saline (PBS). To remove agarose beads, desialylated LDL were centrifuged for 10 min at 2500 rpm and then also dialyzed against PBS.

Naturally occurring atherogenic multiple-modified LDL was isolated from atherosclerotic patients’ plasma by ultracentrifugation as described previously [59,60,61]. The patients had the following characteristics: mean age of patients was 65.0 years (SD = 9.9); body mass index was 25.8 kg/m2 (SD = 3.9); total cholesterol was 240 mg/dL (SD = 28); LDL cholesterol was 148 mg/dL (SD = 23); HDL cholesterol was 69 mg/dL (SD = 11); triglycerides were 113 mg/dL (SD = 27); fasting glucose was 5.1 mmol/L (SD = 0.4); none of the patients had diabetes mellitus. Chemical, physical, and other features at the level of the lipid, protein, and glycoconjugate moieties of naturally occurring atherogenic multiple-modified LDL have been reported previously [60,61,62]. In particular, circulating atherogenic modified LDL differed from native lipoprotein by numerous properties. Among them are small size, high density, increased surface electronegative charge, changes in apo-B tertiary structure, tendency to form self-associates, low concentration of antioxidants, increased susceptibility to oxidation, ability to accumulate lipids in cells, and also the lipid composition. For example, the low level of sialic acid and other neutral carbohydrates, low level of neutral lipids, high level of lysophospholipids, high protein/lipid ratio [61,62].

### 4.2. Monocyte-derived Macrophages

Blood of healthy (with no health problems from the list provided below) volunteers who signed informed consent to participate in the study was used to obtain monocytes by the plastic adhesion methods.

To participate the study, volunteers had to be not regular smokers, not to have cases of acute myocardial infarction among their relatives to the first degree and also not to have following disorders: any manifestations of atherosclerosis, including, but not limited to the acute coronary syndrome, peripheral atherosclerosis, acute myocardial infarction, and CHD (coronary heart disease); impaired glucose tolerance or diabetes mellitus (blood sugar > 110 mg/dL, prescribed sugar-lowering medications, prescribed regular insulin injections); arterial hypertension (diastolic blood pressure > 90 mm Hg and/or systolic blood pressure > 140 mm, prescribed antihypertensive medications); hypercholesterolemia (LDL-cholesterol level > 160 mg/dL, prescribed cholesterol-lowering medications); hypertriglyceridemia (triglycerides > 200 mg/dL, prescribed triglycerides-lowering medications). For monocytes’ isolation, the plasma was removed from the whole blood. The remaining blood was diluted with PBS 1:1. Diluted blood was layered on a density gradient medium of Ficoll and centrifuged to divide mononuclear cells and erythrocytes. Obtained mononuclear cells were placed in the cell culture incubator under 5% CO2 for 2 h at 37 °C. Then, mononuclear cells were washed three times with RPMI-1640 culture medium. Non-adherent cells were removed, and adherent cells were separated and then plated in culture plates (Corning, Corning, NY, USA).

Staining with the anti-CD14-conjugated antibodies was used to determine the purity of the established colony (>95% CD14-positive cells). Staining with Trypan Blue was used to determine the cell viability (>98% in all tests). Cells were then analyzed with the flow cytometry. 

Cells were cultured in RPMI-1640 medium supplemented with 50 ng/mL human M-CSF (PeproTech, Rocky Hill, NJ, USA), 10% of human serum, and 25 ng/mL IL-10 (PeproTech, Rocky Hill, NJ, USA). On the third day, the whole volume of medium was exchanged and, on the 6th day, replaced with a serum-free X-VIVO medium (Lonza Group Ltd., Basel, Switzerland). Then, the serum-free X-VIVO medium was refreshed on day 7, and the obtained monocyte-derived macrophages were used for the tests. On the same day, 50 µg/mL LDL (normalized by total protein measured using Lowry method) or 0.4 μL/mL of latex beads suspension in X-VIVO medium were added and cells were incubated. This amount of LDL and latex beads had no impact on the cell viability. Then cells were harvested, their cholesterol content measured, and RNA was isolated by the RNeasy Plus Mini kit (Qiagen, Hilden, Germany). Each test was performed triply on cells obtained from different volunteers.

### 4.3. Measurement of Intracellular Cholesterol

To assess the intracellular cholesterol content, cells were triply washed in Dulbecco’s phosphate-buffered saline with Ca^2+^ and Mg^2+^, and then lipids were triply extracted by the 3:2 (*v*/*v*) mixture of hexane:isopropanol according to the previously described method [63,64]. After that, the extract was placed in a 96-well plate and dried by the air at room temperature. The resultant precipitate was then diluted with 25 μL of 15 mM sodium cholate in 0.05% Triton X-100 solution (Sigma Chemical Company, St. Louis, MO, USA) and 25 μL of isopropanol and 100 μL of the Monotest solution (Boehringer Mannheim, Germany). The plate was incubated at 37 °C for 30 min, and the optical density was measured in each well at 492 nm with a spectrophotometer (LabSystems, Vantaa, Finland). After lipid extraction, cells were dissolved in 50 μL of 0.2 N NaOH, and the protein contents in each sample were measured using the Lowry method [65].

### 4.4. TRANSFAC and TRANSPATH Databases

RNA-seq libraries were prepared from poly(A)-enriched mRNA using NEBNext Ultra RNA Library Prep kit, amplified for ~12 cycles, and sequenced on a Hi-seq 1500 (Illumina, San-Diego, CA, USA) for 51 cycles. The primary processing of sequencing data was performed using the Torrent Suite. Sequencing quality was evaluated using the FASTQC program. Trimming and removal of technical sequences were performed using the Trimmomatic program. The *H. sapiens* version of GRCh38 was used as the reference sequence. Readings were mapped using the Tophat2 program. To count the number of reads, the htseq-count program was used. The number of raw reads, number of reads after trimming, and the number of aligned reads on the *H. sapiens* genome are shown in Table S1. Principal component analysis was performed and shown in Figure S1. The principal component analysis shows a significant variability of gene expression among the biological replicas. The Limma and RankProd methods were used to identify differentially expressed genes (DEGs) as previously described (Modified LDL Particles Activate Inflammatory Pathways in Monocyte-derived Macrophages: Transcriptome Analysis).

A DNA-binding motifs library obtained from the TRANSFAC^®^ database [66], release 2019.2 (geneXplain, Wolfenbüttel, Germany) (http://genexplain.com/transfac) was used to perform the assay on the transcription factor-binding sites in gene promoters.

Pathway analysis and identification of master regulator genes were performed using a comprehensive signal transduction network based on manually curated TRANSPATH^®^ database release 2019.2 (geneXplain, Wolfenbüttel, Germany) (http://genexplain.com/transpath) [67].

### 4.5. Analysis of Enriched Transcription Factor Binding Sites in Promoters of Differentially Expressed Genes

The Match™ algorithm that helps to search the overrepresented known DNA-binding motifs was used for the analysis of the transcription factor-binding sites in the promoters of the identified differentially expressed genes [68]. Position weight matrices were selected from the TRANSFAC^®^ database and used to determine the motifs.

Then, the frequencies of the transcription factor-binding sites in the promoters of differentially expressed genes (foreground sequence set, denoted as “yes” set) and of genes that were not expressed differentially (background sequence set, denoted as “no” set) were compared. The standard-length promoter sequences, including -1000 bp to +100 bp around the transcription start site, were used for the analysis. Estimating the adjusted *p*-value was used to control the site enrichment error rate (Benjamini–Hochberg correction procedure was used) (adj. *p*-value < 0.01).

The composite module analyst algorithm [69] was used to perform the analysis of the specific combinations of transcription factor-binding sites clustering inside the promoter regions, so-called composite modules, within the promoters of the “yes” and “no” sets. The search object was a composite module, which consists of clusters of sites for a maximum of 10 transcription factors in a sliding window of 200–300 bp that significantly separated the sequences of the “yes” and “no” sets (minimizing Wilcoxon *p*-value).

### 4.6. Identification of Signal Transduction Network

At the previous stage of our investigation, the set of transcription factors was identified. To identify the nodes in the global signal transduction network that potentially regulate the activity of these transcription factors, a comprehensive signal transduction network of human cells (based on the TRANSPATH^®^ database) was used. The main algorithm has been described previously [20,70]. With the use of the formula from our previous study [20], a key-node score for each of such nodes was computed. The identified nodes were ranked in accordance with their potential impact on the activity of the identified transcription factors. As the most significant regulators were highlighted those nodes that showed an ability to specifically regulate the activity of the maximal number of transcription factors from the input list. The algorithm was executed with a maximum radius of 12 steps upstream of each transcription factor in the input set. Applying the algorithm 1000 times to randomly generated sets of input transcription factors of the same set-size was used to control the error rate. The Z-score and FDR (False Discovery Rate) value of ranks were calculated for each of the nodes based on the random runs, as described previously [67]. All nodes with the FDR bellow 0.05 were considered as potential master-regulators. 

### 4.7. Gene Expression Knockdown by Small Interference RNA (siRNA)

Monocyte-derived macrophages were transfected with lipofectamine RNAiMax (Invitrogen, Waltham, MA, USA) following the instructions of the manufacturer for achieving gene knockdown. Target gene-specific siRNA: IL15, EIF2AK3, or F2RL1 (Dallas, TX, USA, Santa Cruz Biotechnology) or 50 nM of a control scrambled siRNA were used for the transfection.

## 5. Conclusions

We have shown that not less than 12 signaling pathways are likewise regulated by the interaction of macrophages with the multiply modified atherogenic naturally occurring LDL and with latex beads. We demonstrated that modified LDL triggered the genetic regulation characteristic of phagocytosis stimulation. Modified lipoprotein particles may stimulate phagocytosis due to the formation of self-associates that are taken up through non-specific phagocytosis rather than specific LDL receptor. 

At least three genes, *F2RL1*, *EIF2AK3*, and *IL15*, were determined to encode inflammatory molecules and to be associated with signaling pathways identified in the present study. The upregulation of these genes was noted in response to the interaction of modified LDL with macrophages. Furthermore, knockdowns of *EIF2AK3* and *IL15* completely suppressed the accumulation of cholesterol in macrophages. Hence, the upregulation of *EIF2AK3* and *IL15* genes promoted the accumulation of cholesterol. These data confirmed our hypothesis based on the following views: LDL particles acquire atherogenic properties that contribute to the multiple modifications. These changes are accompanied by the formation of self-associates; that stimulate phagocytosis due to their size. This resulted in the secretion of pro-inflammatory molecules, that, in turn, cause or at least contribute to the accumulation of intracellular cholesterol. This data makes obvious that the primary in this sequence of events is not the accumulation of cholesterol but an inflammatory response and also explain the relations of this crucial for atherosclerosis development processes.

## Figures and Tables

**Table 1:** Signaling pathways regulated by the interaction of macrophages with modified LDL and latex beads. Native LDL was excluded from the consideration because it does not cause lipid accumulation. Signaling pathways adjustable unidirectionally in all four types of modifications are highlighted in green; signaling pathways, the direction of regulation of which coincides with the three types of modifications are highlighted in cyan color; signaling pathways coinciding only in the case of two modifications are highlighted in yellow.

Modification	Regulation	Signaling Pathway
Naturally Occurring	up	neurotrophic signaling TLR2-mediated signaling TLR9 pathway VEGF-A pathway
	down	Aurora-B cell cycle regulation Cdc20 deubiquitination Cdc20 ubiquitination cyclinB1 ubiquitination ---> anaphase onset Fzr1 ---> cyclin B1 degradation Metaphase to Anaphase transition securin degradation Usp44 ---> Cdc20
Desialylation	up	Apo2L pathway Fas pathway insulin ---> ERK neurotrophic signaling TLR9 pathway
	down	Aurora-B cell cycle regulation Cdc20 deubiquitination Cdc20 ubiquitination cyclinB1 ubiquitination ---> anaphase onset Fzr1 ---> cyclin B1 degradation Metaphase to Anaphase transition securin degradation Usp44 ---> Cdc20
Acetylation	up	alpha IIb beta3 ---> Rac1 alpha IIb beta3 pathway angiotensin II ---> DAG, CaMKII Aurora-A activation, substrates, and degradation Aurora-A cell cycle regulation B-cell antigen receptor pathway BCR ---> ERK BDNF ---trkB---> MAPK cascade beta-glucan ---> AKT-1 Fas pathway G-alpha-q ---> arachidonic acid, ERK insulin ---> ERK insulin pathway insulin ---Shc---> MAPK cascade KSR scaffold complex neurotensin pathway neurotrophic signaling p38 pathway p53 pathway PDGF A ---> ERK PDGF B ---> ERK PKC ---> ERK1, ERK2 POSH ---> JNK1, JNK2 PRL ---Src, FAK1---> ERK RANKL ---> p38 RANKL pathway Tiam1 ---> p38alpha TLR2 ---Rac1--->AKT TLR2-mediated signaling TLR3 pathway TLR9 pathway tuberin pathway VEGF-A pathway
	down	Aurora-B cell cycle regulation
Oxidation	up	AR pathway Fas pathway HIF-1alpha pathway IRAK-1 ---MKK3---> TNF p38 pathway p53 pathway PKC ---> ERK1, ERK2 RANKL ---> p38 RANKL pathway TLR2-mediated signaling TLR3 pathway TLR9 pathway
	down	-

**Table 2:** Effect of gene knockdown on cholesterol accumulation in cultured macrophages.

Knock-downed Gene		Number of Experiments	Relative Content of Intracellular Cholesterol (SD)	*p* (*t*-test)		*p* (Wilcoxon–Mann–Whitney test)	
				vs. ‘Control’	vs. ‘+ LDL’	vs ‘Control’	vs. ‘+ LDL’
*EIF2AK3* (-)	Control	6	1.00 ± 0.06 (0.35)	-	-	-	
	+ LDL	6	1.36 ± 0.09 (0.58)	0.002	-	0.001	-
	EIF2AK3 ^(-)^ + LDL	6	0.98 ± 0.07 (0.43)	0.86 NS	0.002	0.53 NS	0.007
*IL15*	Control	4	1.00 ± 0.10 (0.58)	-	-	-	
	+ LDL	4	1.29 ± 0.07 (0.19)	0.038	-	0.038	-
	IL15 ^(-)^ + LDL	4	0.89 ± 0.09 (0.49)	0.35 NS	0.031	0.35 NS	0.011
*F2RL1*	Control	4	1.00 ± 0.07 (0.37)	-	-	-	
	+ LDL	4	1.29 ± 0.09 (0.46)	0.014	-	0.016	-
	F2RL1 ^(-)^ + LDL	4	1.37 ± 0.13 (0.71)	0.018	0.64 NS	0.019	0.52 NS

Control, macrophages with the addition of control siRNA that does not lead to gene knockdown. + LDL, atherogenic naturally occurring LDL (50 µg protein/mL) was added to the ‘Control’. NS = Not significant.

## Supplementary Materials

The following are available online at https://www.mdpi.com/1422-0067/21/8/2716/s1.
