# Supplementary material for: Signaling Pathways Potentially Responsible for Foam Cell Formation: Cholesterol Accumulation or Inflammatory Response—What is First?
Source: Int J Mol Sci. 2020 Apr 14;21(8):2716. doi: 10.3390/ijms21082716 (PMC7216009; doi:10.3390/ijms21082716)
Supplement: Supplementary file 1 [file ijms-21-02716-s001.zip › suppl_3_description.docx]

Figure S3. Neurotrophic signaling. The red frame shows the components of the signaling pathway that are associated with the proteins encoded by the genes F2RL1 (PAR2), EIF2AK3 (PERK) and IL15. The red nodes represent potential regulators found by the algorithm of network analysis, the blue nodes represent the target proteins that were used as the starting point of the algorithm, the green nodes are the intermediate proteins of the signal transduction between regulators and the target proteins.
