# Supplementary material for: Signaling Pathways Potentially Responsible for Foam Cell Formation: Cholesterol Accumulation or Inflammatory Response—What is First?
Source: Int J Mol Sci. 2020 Apr 14;21(8):2716. doi: 10.3390/ijms21082716 (PMC7216009; doi:10.3390/ijms21082716)
Supplement: Supplementary file 1 [file ijms-21-02716-s001.zip › Table S1.docx]

| sample | # raw reads | # reads after trimming | # aligned reads on *H.sapiens* genome |
| --- | --- | --- | --- |
| 71_7_Control | 16,212,839 | 12,551,577 | 12,361,146 |
| 72_7_NativeLDL | 18,459,275 | 14,329,514 | 14,155,984 |
| 73_7_AtherogenicLDL | 21,182,231 | 16,492,353 | 16,262,456 |
| 74_7_DesialylatedLDL | 17,208,522 | 13,390,601 | 13,214,422 |
| 75_7_AccLDL | 21,626,461 | 16,692,360 | 16,477,958 |
| 76_7_Larex | 22,713,208 | 17,659,498 | 17,357,708 |
| 77_OxLDL | 22,788,808 | 18,056,556 | 17,768,173 |
| 81_8_Control* | 16,512,328 | 13,487,610 | 3,643,108 |
| 82_8_NativeLDL | 20,490,105 | 15,809,057 | 15,574,580 |
| 83_8_AtherogenicLDL | 31,037,036 | 23,846,948 | 22,828,605 |
| 84_8_DesialyatedLDL | 22,090,141 | 17,158,315 | 16,450,787 |
| 85_8_AccLDL | 19,446,164 | 14,945,815 | 14,285,897 |
| 86_Larex | 24,163,976 | 18,639,516 | 17,904,938 |
| 87_8_OxLDL | 27,343,532 | 20,809,534 | 20,442,170 |
| 91_9_Control | 23,084,072 | 17,697,264 | 17,398,608 |
| 92_9_NativeLDL | 33,867,889 | 26,028,128 | 25,544,365 |
| 93_9_AtherogenicLDL | 19,491,598 | 14,995,870 | 14,758,588 |
| 94_9_DesialylatedLDL* | 2,909,764 | 2,248,630 | 2,216,912 |
| 95_9_AccLDL | 18,483,551 | 14,310,504 | 14,085,654 |
| 96_9_Larex | 20,109,877 | 15,604,625 | 15,376,816 |
| 97_9_OxLDL | 15,233,226 | 11,793,289 | 11,620,245 |

Table S1. Number of reads aligned on H.sapiens genome. * - Samples 81 and 94 were excluded from the further analysis.
