# Supplementary figures and images for: Signaling Pathways Potentially Responsible for Foam Cell Formation: Cholesterol Accumulation or Inflammatory Response—What is First?
Source: Int J Mol Sci. 2020 Apr 14;21(8):2716. doi: 10.3390/ijms21082716 (PMC7216009; doi:10.3390/ijms21082716)

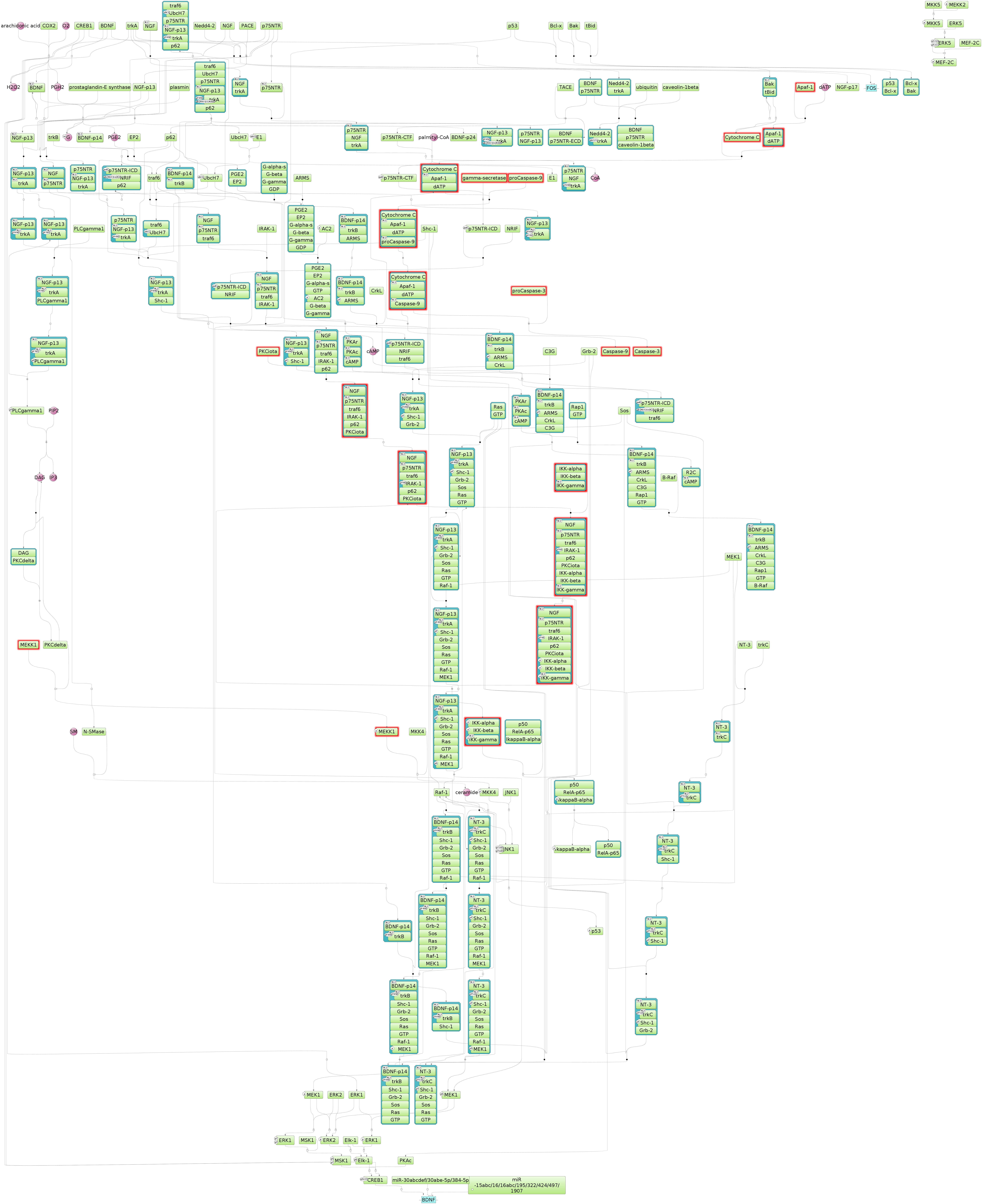

Supplement: Supplementary file 1 [file ijms-21-02716-s001.zip › Figure S2.png]

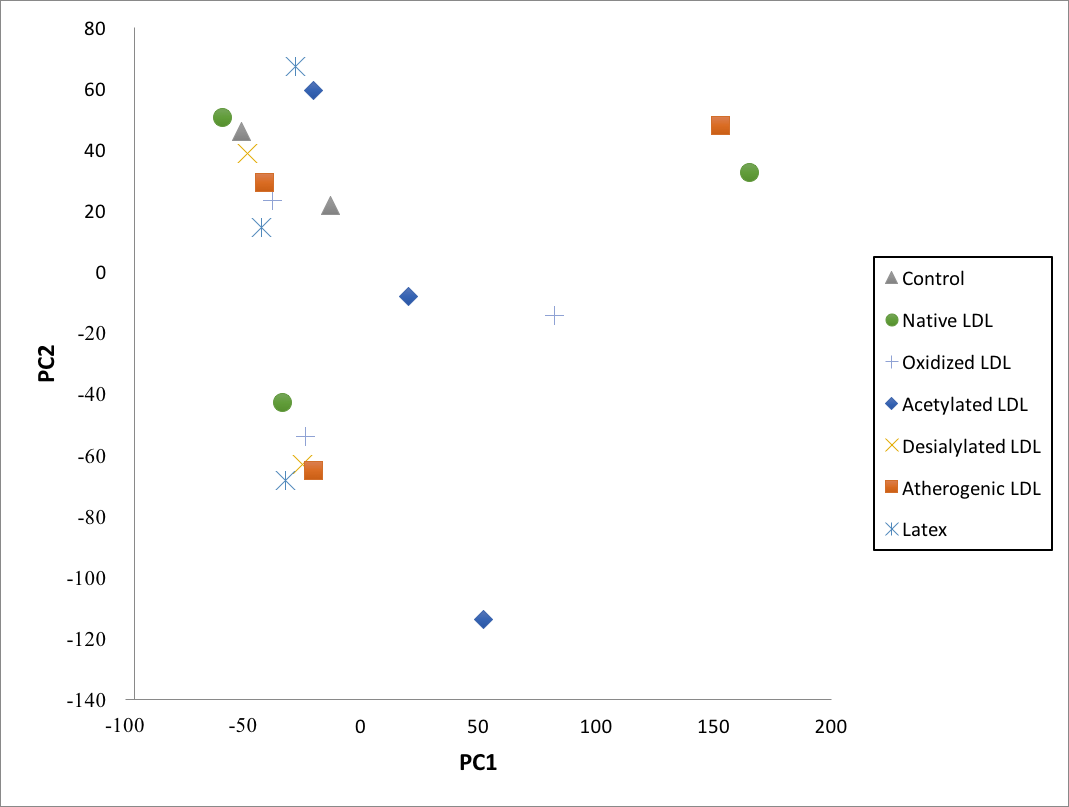

Supplement: Supplementary file 1 [file ijms-21-02716-s001.zip › FigureS1.png]
